# Identification of Important Modules and Biomarkers That Are Related to Immune Infiltration Cells in Severe Burns Based on Weighted Gene Co-Expression Network Analysis

**DOI:** 10.3389/fgene.2022.908510

**Published:** 2022-06-09

**Authors:** Zexin Zhang, Yan He, Rongjie Lin, Junhong Lan, Yueying Fan, Peng Wang, Chiyu Jia

**Affiliations:** ^1^ Department of Burns and Plastic and Wound Repair Surgery, Xiang’an Hospital of Xiamen University, School of Medicine, Xiamen University, Xiamen, China; ^2^ Department of Burns and Plastic and Cosmetic Surgery, The Ninth Affiliated Hospital of Xi’an Jiaotong University, Xi’an, China; ^3^ Department of Orthopedics, The 900th Hospital of Joint Logistic Support Force, Fuzhou, China

**Keywords:** immunosuppression, burns, WGCNA, LASSO, GSVA, CIBERSORT, prognostic biomarker

## Abstract

**Background:** Immunosuppression is an important trigger for infection and a significant cause of death in patients with severe burns. Nevertheless, the prognostic value of immune-related genes remains unclear. This study aimed to identify the biomarkers related to immunosuppression in severe burns.

**Methods:** The gene expression profile and clinical data of 185 burn and 75 healthy samples were obtained from the GEO database. Immune infiltration analysis and gene set variation analysis were utilized to identify the disorder of circulating immune cells. A weighted gene co-expression network analysis (WGCNA) was carried out to select immune-related gene modules. Enrichment analysis and protein–protein interaction (PPI) network were performed to select hub genes. Next, LASSO and logistic regression were utilized to construct the hazard regression model with a survival state. Finally, we investigated the correlation between high- and low-risk patients in total burn surface area (TBSA), age, and inhalation injury.

**Results:** Gene set variation analysis (GSVA) and immune infiltration analysis showed that neutrophils increased and T cells decreased in severe burns. In WGCNA, four modular differently expressed in burns and controls were related to immune cells. Based on PPI and enrichment analysis, 210 immune-related genes were identified, mainly involved in T-cell inhibition and neutrophil activation. In LASSO and logistic regression, we screened out key genes, including *LCK, SKAP1* and *GZMB,* and *LY9*. In the ROC analysis, the area under the curve (AUC) of key genes was 0.945, indicating that the key genes had excellent diagnostic value. Finally, we discovered that the key genes were related to T cells, and the regression model performed well when accompanied by TBSA and age.

**Conclusion:** We identified LCK, SKAP1, GZMB, and LY9 as good prognostic biomarkers that may play a role in post-burn immunosuppression against T-cell dysfunction and as potential immunotherapeutic targets for transformed T-cell dysfunction.

## Introduction

There are 180,000 people who die as a result of burns, with 47 percent of those fatalities linked to infection-related complications. Infections in severe burns are often caused by an overactive inflammatory response and immunosuppression. In severe burns, adaptive immune functions represented by T cells are suppressed and inflammatory responses represented by neutrophils and dendritic cells are activated (Miller et al., 2007; Sood et al., 2016; Hampson et al., 2017). Impaired skin and intestinal mucosal barriers are exposed to pathogens, and the disorder of peripheral blood cells results in a low response to pathogens (Neely et al., 2004; Wrba et al., 2017). All of that mentioned above lead to uncontrollable infection and death (Fitzwater et al., 2003).

Immunosuppression in severe burns is considered to be significantly correlated with prognosis. In the early stages of burns, PAMP-mediated innate immune translation was enhanced, such as macrophages, dendritic cells, and neutrophils being recruited to the injured site to clear necrotic tissue. Subsequently, the adaptive immune response is impaired, such as Th cell subtype imbalance, where Th1 cells are inhibited and Th2 cells are activated, resulting in immunosuppression. Inflammatory factors, cytokines, and immune cells have a significant prognostic value in severe burns (Hur et al., 2015; Osuka et al., 2019). Previous studies have explored the prognostic value of platelets, inflammatory factors, immune-related cytokines, and scoring scales. However, the accuracy and clinical practicability need to be improved. These prognostic factors cannot explain the disturbance of homeostasis after severe burns, especially immunosuppression (Hur et al., 2015; Lip et al., 2019; Geng et al., 2020). Alterations in gene expression profiles underlie disease development and can reflect changes in homeostasis from a pathophysiological perspective, explaining the mechanisms that affect prognosis and providing therapeutic targets for subsequent studies (Gaetani et al., 2019; Zou and Wang 2019). In addition, gene detection is convenient, economical, and has a strong stability in the application of prognosis. Genes have been used as biomarkers to model the prognosis of a variety of diseases, showing strong prognostic power. However, their application to burns is rare (Sandquist and Wong 2014; Foth et al., 2016; Gavrielatou et al., 2020). Therefore, it is meaningful to explore immune-related genes in severe burns for revealing their value as prognostic biomarkers and immune therapeutic targets.

This study is a large population-based prognostic study involving 185 burn patients. We investigated the relationship between gene expression profiles and the prognosis of severe burns using machine learning algorithms (Newman et al., 2015). The disorder of immune cells in severe burns was investigated by CIBERSORT and gene set variation analysis (GSVA) (Hänzelmann et al., 2013). The genes related to the disorder of immune cells were identified in WGCNA. We used the Least Absolute Shrinkage and Selectionator operator (LASSO) and logistic regression to create a prognostic model for immune-related genes. Additionally, we identified the correlation between cellular subtypes and genes, which were associated with immune abnormalities following severe burns. The research aimed to provide a certain basis and reference value for revealing the prognostic value of genes associated with immunosuppression.

## Methods

### Acquisition of RNA Data

**FIGURE 1:**
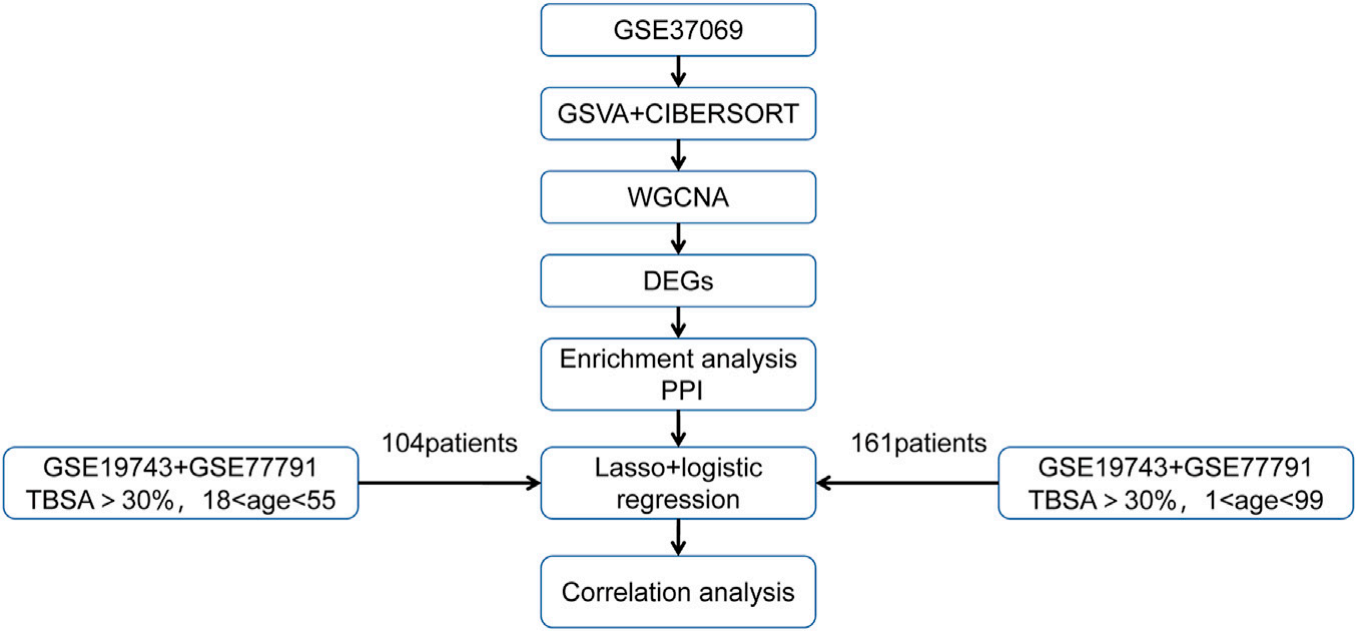
A graphical summary of the research design.

We downloaded three microarray expression profiles and clinical data of severe burns (GSE19743, GSE77791, and GSE37069) from the GEO database (http://www.ncbi.nlm.nih.gov/geo/). Patients >18 years or <55 years and sampling time between 280 and 705 h were selected to remove the influence of age and sampling time.(Table 1). Meanwhile, we downloaded clinical information (survival, burn area, sampling time, and age) from three datasets. GSE37069 was utilized to screen immune- and prognostic-related genes between burn and control (Supplementary Data Sheet S1), GSE77791 and GSE19743 were utilized to be the training cohort and validating cohort, between survival and non-survival, respectively (Supplementary Data Sheets S2, S3). There was no need for patients’ consent and ethical approval as all data were taken from public databases. The experimental procedure was as shown in Figure 1.

**TABLE 1:** Clinical data of burn patients and health controls in GSE37069 and GSE19743.

Group (burn)	Sex			Age	Time of sampling
	N	Male	Female		
GSE19743	28	24	4	37.61 ± 7.98	439.28 ± 117.86
GSE37069	81	57	24	37.41 ± 10.45	411.92 ± 124.76
*P*	0.109			0.271	0.311

Group (health)	Sex			Age	Time of sampling
	N	Male	Female		
GSE19743	25	14	11	30.21 ± 8.16	—
GSE37069	37	17	20	32.59 ± 11.03	—
*P*	0.709			0.364	—

### Data Processing

The expression profiles were processed by the package of R software, “*Affy*.” The background correction of the expression value and normalization of the expression profile data were performed, including conversion of original data format, the supplement of missing value, background correction, and data standardization by using the Quantile method.

### GSVA

GO term related to immune cells was selected from the GSEA website (http://www.gsea-msigdb.org/gsea/index.jsp). The GSVA analysis was performed between burns and healthy controls in GSE37069 by the “*GSVA*” package in R software. Unqualified samples were removed prior to the variance analysis. The result of GSVA was analyzed by the R package “limma” to calculate the differences in enrichment results between severe burns and healthy controls.

### Immune Infiltration Analysis

“*CIBERSORT*” is a machine learning algorithm that can analyze the proportion of immune cells from RNA-seq (Newman et al., 2015). We downloaded the expression profile of GSE37069, GSE19743, and GSE77791 to select 81, 28, and 76 burn patients and 37, 25, and 13 healthy controls, respectively, and performed an immune infiltration analysis by using the “*CIBERSORT*” package in R software. Cell subtypes with *p* < 0.05 in three gene sets were considered as key immune cells (KICs) for further analysis. Next, the “*ggplot2*” package in the R software was used to visualize the different proportion of KICs between burn patients (Group2) and healthy controls (Group1).

### WGCNA

WGCNA could construct a scale-free distribution network by using soft power to classify genes with the same expression trend and analyze the correlation between genes and traits (Langfelder and Horvath 2008). We performed WGCNA on the gene set GSE37069 by using the “*WGCNA*” package in the R software and identified gene modules associated with key immune cells. The intersection of GSVA and immune infiltration analysis are defined as clinical traits to identify immune-related gene modules.

### Differential Expression Analysis

We utilized the “*LIMMA*” package in R software (version 4.0.5) to analyze DEGs in GSE19743, GSE77791, and GSE37069 datasets (FDR<0.05, |logFC|>1) and took the intersection of genes in GSE19743, GSE77791, GSE37069, and immune-related modules.

### Enrichment Analysis and PPI

We performed an enrichment analysis of differently expressed genes in the immune-related gene modules.(modules with differential genes more than 20 were selected). We used the DAVID6.8 online tool (https://david.ncifcrf.gov) to perform the enrichment analysis of Kyoto Encyclopedia of Genes and Genomes (KEGG) and Gene Ontology (GO) and the “*ggplot2*” package in R software was used to draw a bubble chart. We constructed the interaction network between the enrichment results (FDR<0.05). We selected immune-related genes in the enrichment analysis and constructed the PPI network which was visualized by Cytoscape and hub genes selected by MCODE.

### LASSO and Logistic Regression

In GSE19743 and GSE77791 (age 18–55, TBSA >30%, sampling time 280–705 h), we performed a LASSO regression analysis by using the “l*asso*” package of R software to screen hub genes. The hub genes were analyzed by ROC curves and the variables with AUC>0.6 were selected for a logistic regression to establish the regression model. The regression model used GSE77791 as the training cohort and GSE19743 as the validation cohort. The nomogram plot was utilized to calculate the risk score, and the model was evaluated by the ROC curve and calibration curve.

### Multidimensional Validation

To explore the effects of burn area and age on the model, we reincorporated 161 patients of GSE19743 and GSE77791 to validate the prognostic ability of the key genes. Burn area, age, and key genes were included in for modeling. Regression models were constructed by random grouping (70% training cohort and 30% validation cohort). A nomogram was drawn to calculate the risk score, and the ROC curve was used to evaluate the accuracy.

### Correlation Analysis

A correlation analysis was performed between key immune cell subtypes and genes, LCK, SKAP1, GZMB, and LY9. The patients were divided into two groups according to the risk score (high risk: score>0.5, low risk: score<0.5). The prognosis, age, gender, TBSA, and inhalation injury were contrasted between the high- and low-risk groups.

### Validation of Key Gene Expression

To further verify the prognostic value of the key genes, we verified the expression profiles of key genes in death and survivors of burn patients in independent cohorts, GSE19743 and GSE77791. We converted the fluorescence values of each sample to log2 and averaged the different probes of the same gene.

### Statistics Method

Variables are represented by mean±σ. Comparisons of data were made by using the Chi-square test for categorical data or Student’s t-test for normalized quantitative data as appropriate. The multiple logistic regression model was utilized to examine the relationship between mortality and variables. The criterion variable was death as the outcome. The explanatory variables included age, gender, %TBSA, and expression of genes. ROC and calibration curves were utilized to process prognostic ability logistic models.

## Result

### Acquisition of RNA Data

We selected 28, 76, and 81 burn patients with a control group of 25, 13, and 37 health controls in the GSE19743, GSE77791, and GSE37069 datasets. GSE19743 and GSE77791 (training and validating cohorts) had 104 severe burns, with 81 survivors and 23 deaths (Table 2).

**TABLE 2:** Clinical data of burn patients in GSE77791 and GSE19743.

Group	Sex			Age	TBSA		Time of sampling (GSE19743)
	N	Male	Female		Severe (30–49)	Major (49–100)	
Death	23	19	4	40.73 ± 7.67	3	20	422.24 ± 122.32
Survival	81	70	11	40.67 ± 10.74	21	60	393.33 ± 113.19
*P*	0.147			0.974	0.001		0.544

### GSVA

**FIGURE 2:**
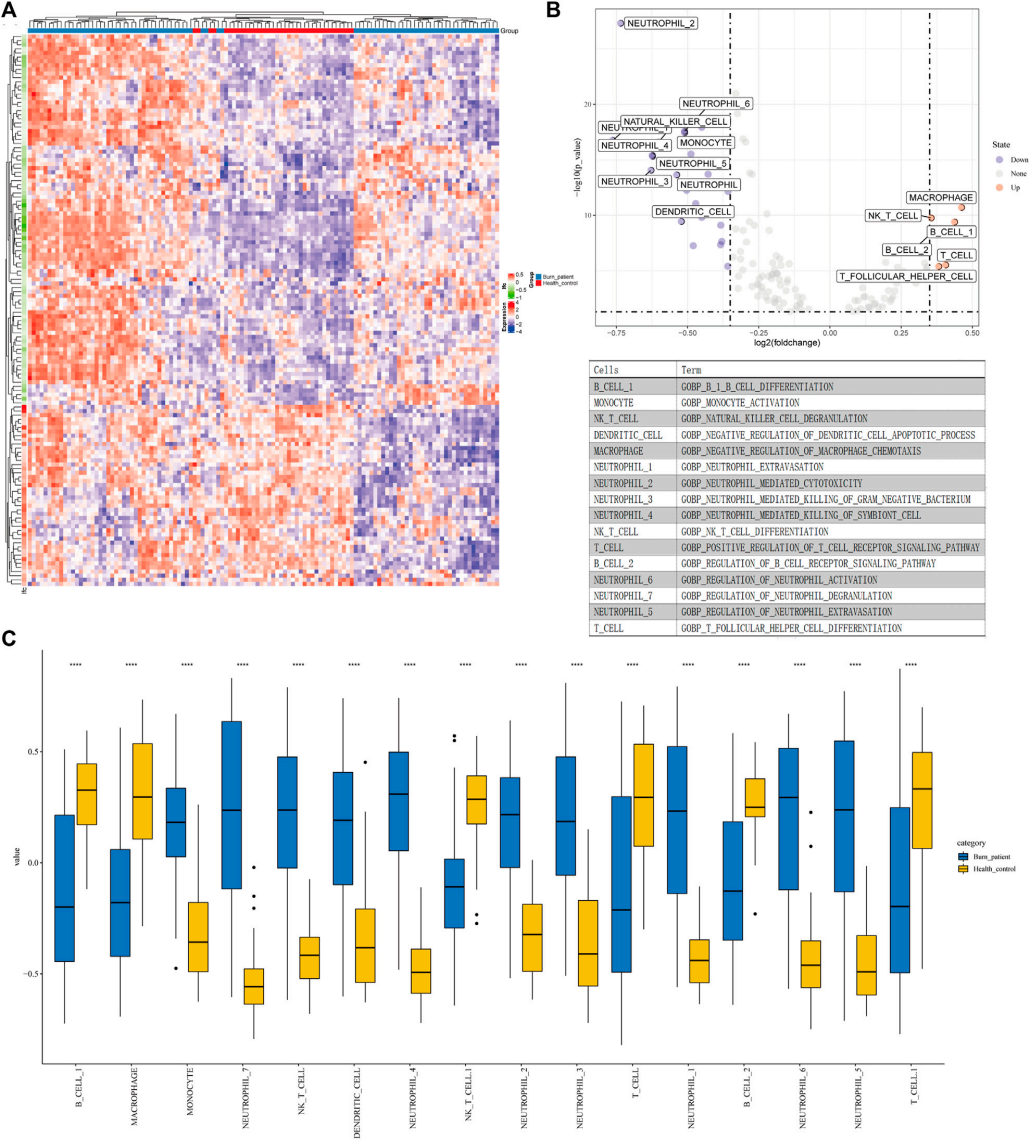
Results of GSVA and CIBERSORT **(A)** Heat map of 203 GO terms between severe burns and controls. **(B,C)** Different terms of GSVA with |logFC|>0.35, *p* < 0.05.

We selected 203 immune-related GO terms which had significant differences in immune-related pathways between normal and severe burns (Figure 2A). Adj. p < 0.05, |logFC| > 0.35 are considered to be different GO terms in the differential analysis (Figure 2B). In severe burns, the enrichment score of neutrophil, dendritic, monocyte, and NKT cell-related pathways were increased while T cells, B cells, and macrophages were decreased (Figure 2C).

### Immune Infiltration Analysis

**FIGURE 3:**
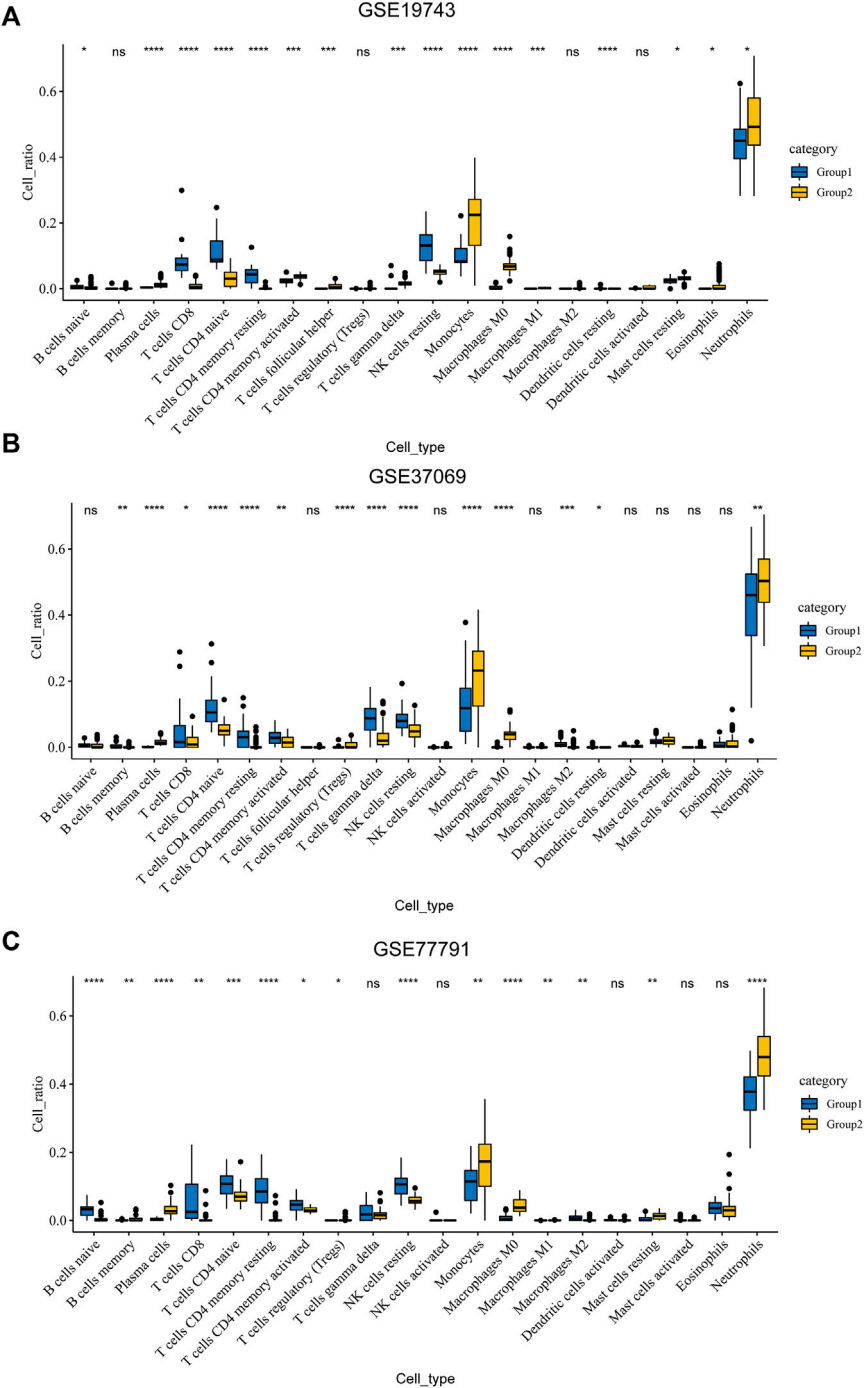
Results of CIBERSORT. **(A)** Different ratios of the 22 immune cells between severe burns and controls in GSE19743. **(B)** Different ratios of the 22 immune cells between severe burns and controls in GSE37069. **(C)** Different ratios of the 22 immune cells between severe burns and controls in GSE77791.

An analysis of immune infiltration showed that plasma cells, T cells CD8, T cells CD4 naive, T cells CD4 memory resting, T cells CD4 memory activated, NK cells resting, monocytes, macrophages M0, dendritic cells resting and neutrophils were KICs (Figures 3A–C). We took the intersection of GSVA and CIBERSORT results. All seven kinds of immune cells, which were utilized for WGCNA, had a significant difference between burns and healthy controls.

### WGCNA

**FIGURE 4:**
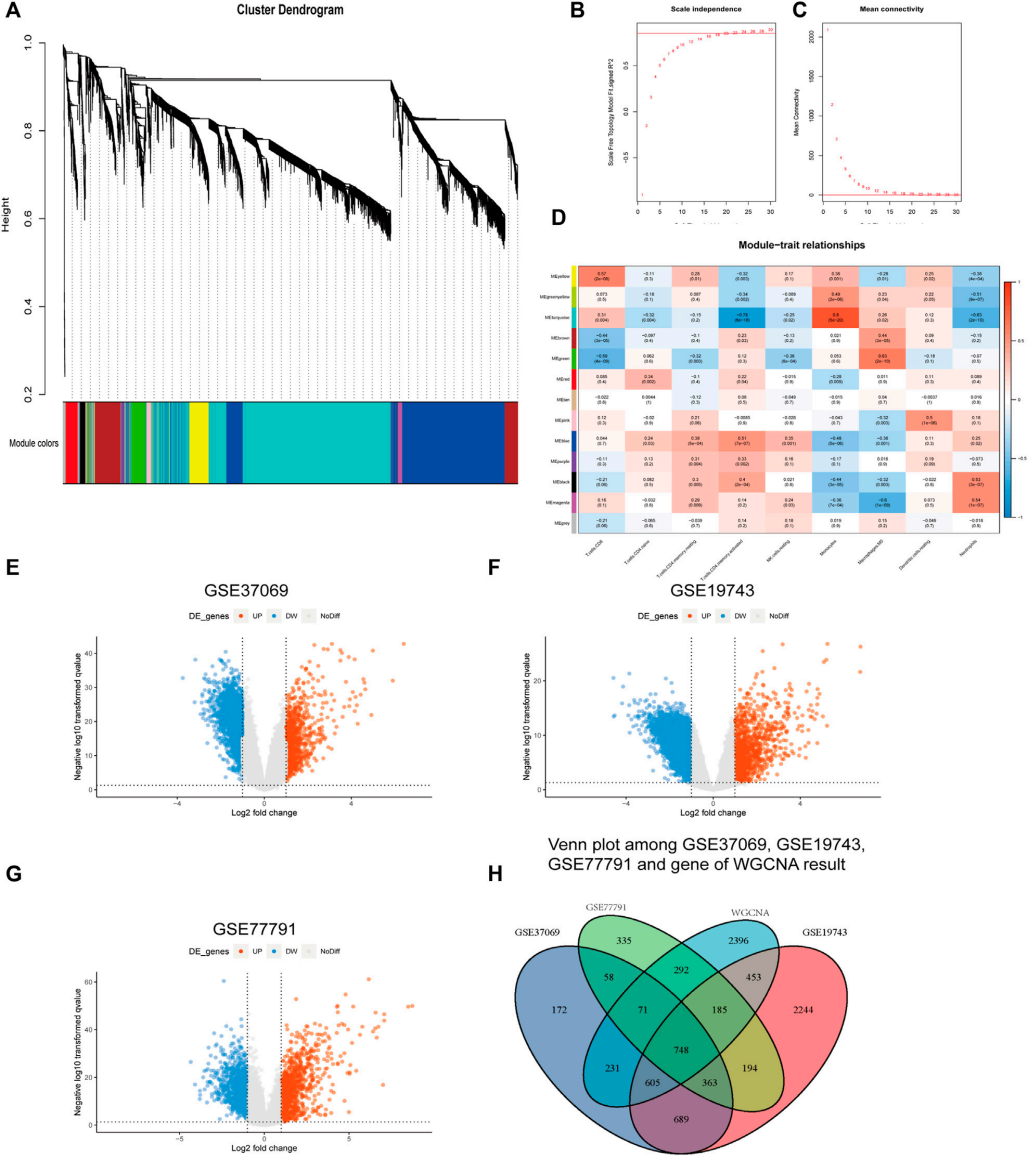
Results of WGCNA and different expression analysis. **(A)** Genes in GSE37069 were divided into 12 modules in different colors. Genes in the same color module have similar expression patterns. **(B,C)** Soft power of WGCNA. **(D)** Correlation genes and immune cells. The ordinate is the gene module; the abscissa is the cell type. Blue are negative correlations and red are positive correlations. **(E–G)** The ordinate is the -log_10_ *p* value; the abscissa is the logFC. **(H)** Intersection of genes in GSE19743, GSE37069, GSE77791, and immune cell-related genes in WGCNA.

Genes in GSE37069 were divided into twelve modules (Figure 4A). The soft power was 22 (R > 0.85) (Figures 4B,C). Yellow, turquoise, green, and blue modules were related to the proportion of immune cells and named immune-related modules (correlated to T cells, *p* < 0.05, correlation coefficient > 0.5) (Figure 4D).

### Differential Expression Analysis

We obtained 2,937, 5,481, and 3,233 differential expression genes from GSE37069, GSE19743, and GSE77791 (|logFC|>2, *adj. p* < 0.05) and there are 748 differential expression genes in immune-related modules (Figures 4E–G).

### Enrichment Analysis and PPI

**FIGURE 5:**
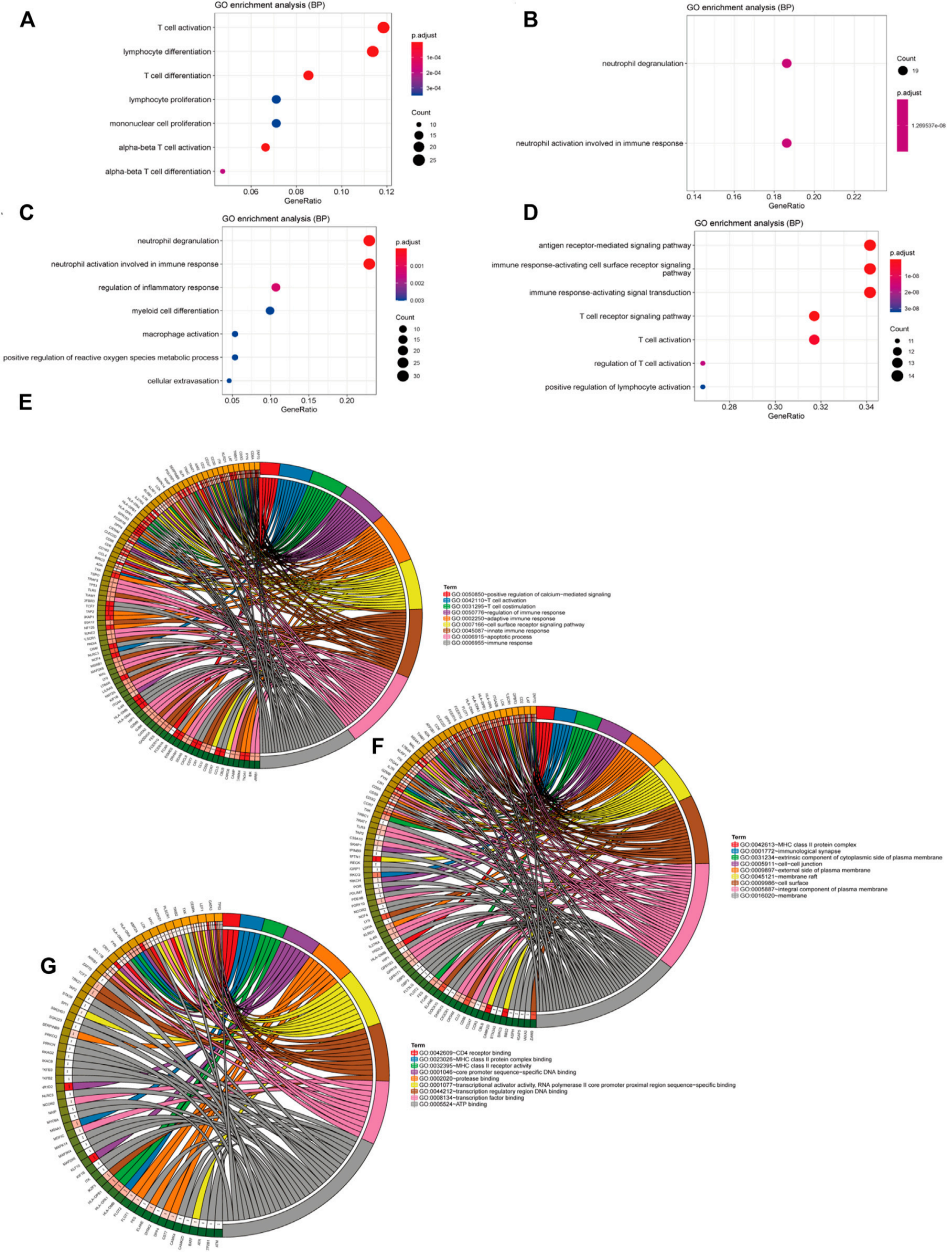
Results of the enrichment analysis. **(A–D)** The ordinate is the pathway name and the abscissa is the proportion of genes in the pathway. The redder the circle, the bigger the *p* value. **(A)** Enrichment analysis of genes in the blue module in WGCNA, **(B)** in green, **(C)** in turquoise, and **(D)** in yellow. **(E–G)** Results of the enrichment analysis with 210 immune-related genes that we got from the pathway in the enrichment analysis in the four gene modules.

**FIGURE 6:**
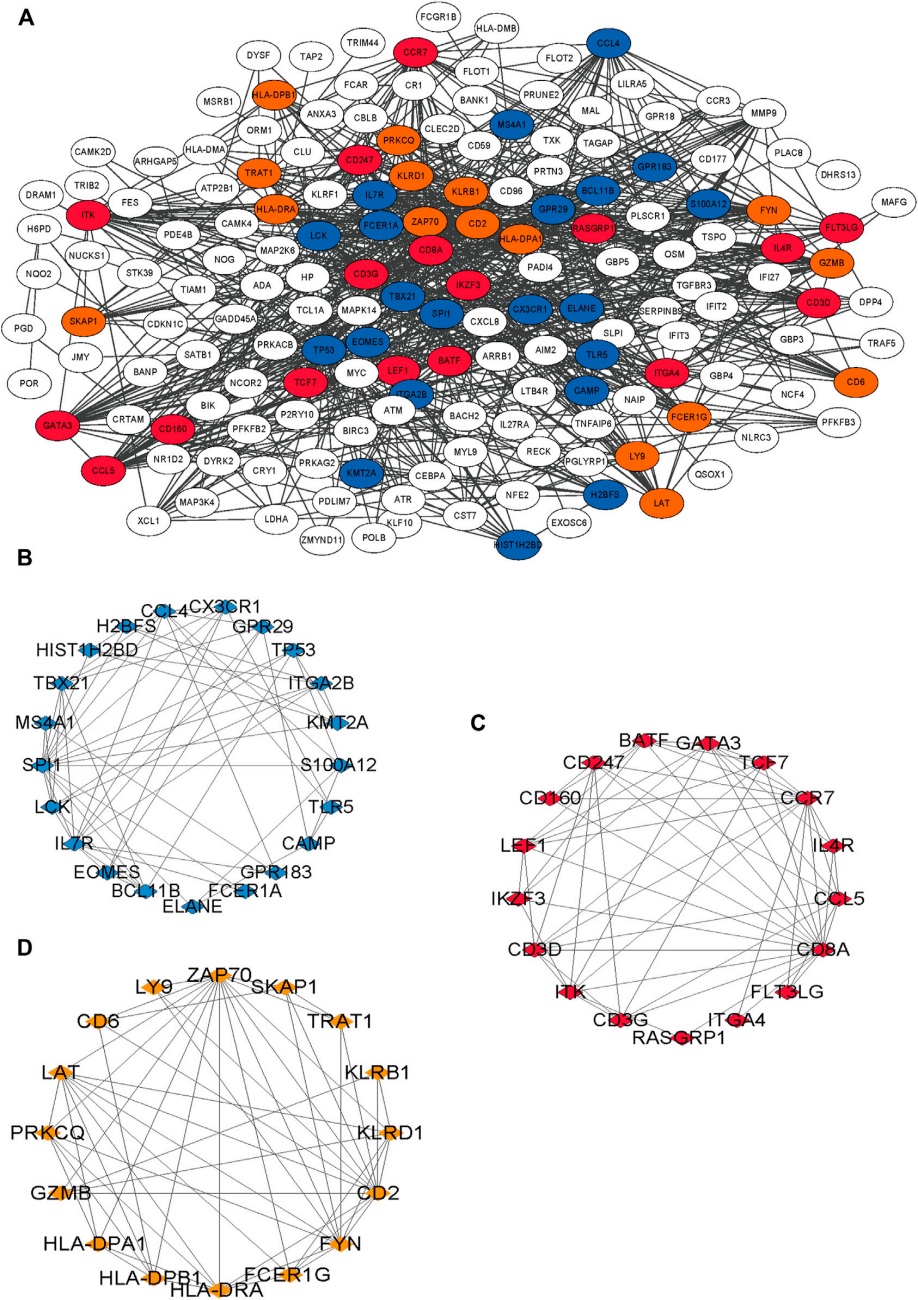
Results of PPI network of 210 immune-related genes. **(A)** The interaction of 210 genes, different colors means different interaction groups. **(B–D)** Blue is module 1, orange is 2 and red is 3.

The blue module was mainly enriched in T-cell activation, lymphocyte differentiation, and T-cell differentiation (Figure 5A). The green module was mainly enriched in neutrophil degranulation and neutrophil activation involved in immune response (Figure 5B). Turquoise was mainly enriched in neutrophil degranulation and neutrophil activation involved in immune response and regulation of inflammatory response (Figure 5C). Yellow was mainly enriched in the antigen receptor-mediated signaling pathway, immune response-activating cell surface receptor signaling pathway, and immune response-activating signal transduction pathway (Figure 5D). A total of 210 immune-related genes were found in the aforementioned immune-related enrichment results, which were mainly related to the function and cell structure of immune-related genes such as T cells, immune response, MHC II class protein complex, CD4 receptor, and Ca^2+^ signal pathway (Figures 5E–G). Three core modules were selected in the MCODE module, which were marked by blue, red, and orange, with a total of 53 hub genes (Figures 6A–D).

### LASSO and Logistic Regression

**FIGURE 7:**
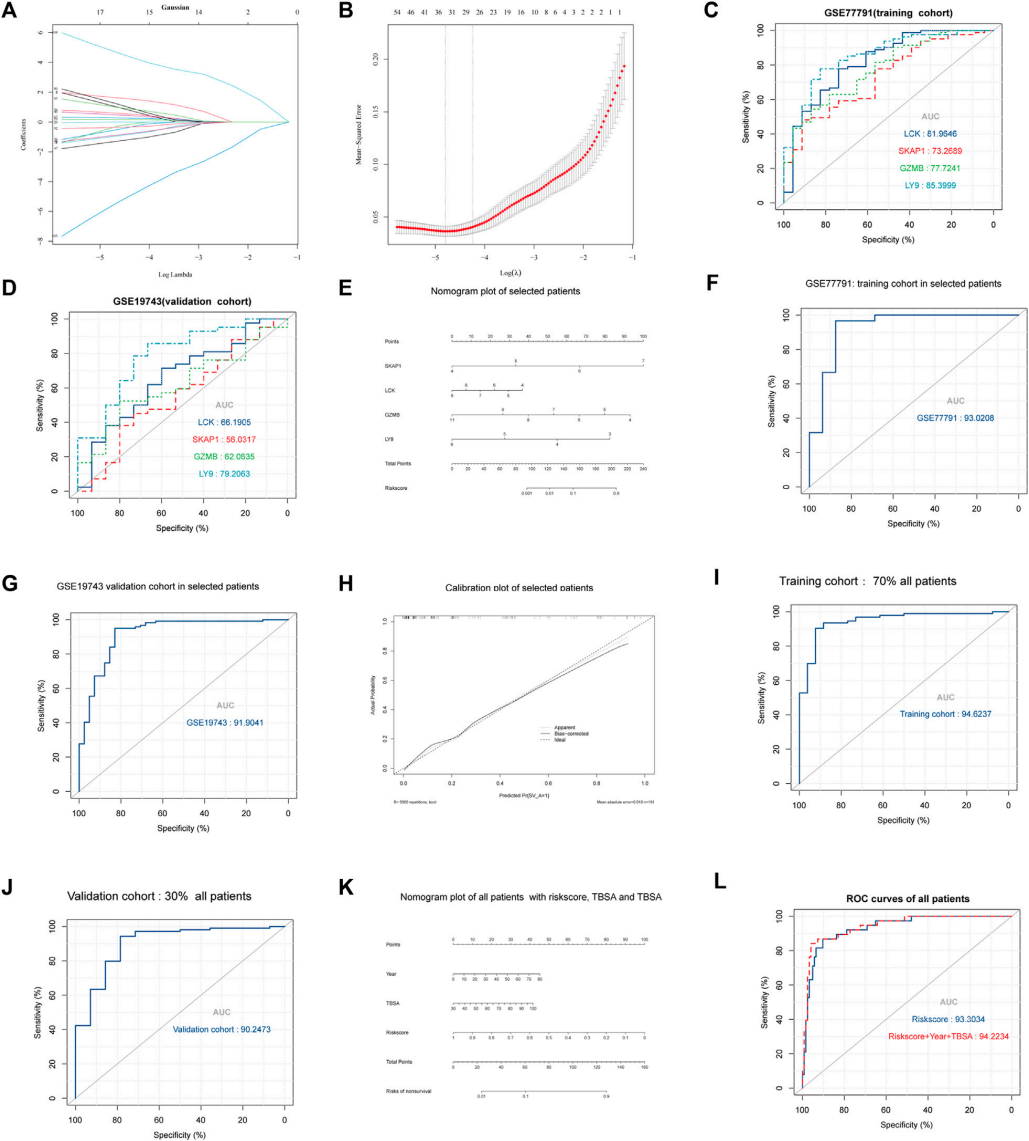
Results of regression, multidimensional verification, and correlation between key genes and immune cells. **(A,B)** The ordinate is the correlation coefficient between gene expression and prognosis. λ was utilized to screened genes and the number aforementioned is the number of genes. **(C)** AUC of the four key genes in GSE77791. The larger the area under the curve, the stronger the prediction ability. **(D)** AUC of the four key genes in GSE19743. **(E)** Nomogram plot of logistic regression which can be utilized to calculate the risk score between severe burns and controls. **(F)** AUC of logistic regression with selected patients (18 < age<55, TBSA>30, 280 h < sample times <706) in the training cohort (GSE77791). **(G)** AUC of logistic regression with selected patients (18 < age<55, TBSA>30%, 280 h < sample times <706) in the validation cohort (GSE19743). **(H)** Calibration curve of logistic regression. The closer bias-corrected curve and ideal curve are, the better predictive regression model is. **(I,J)** Selected patients (*N* = 104, 18 < age<55, TBSA>30, 280 h < sample times <706) were divided into two cohorts in randomly to train and validate regression model, the training cohort had 70% patients, and the validation cohort had 30%. **(K,L)** All patients (*N* = 161, 1 < age<99, TBSA>30%, 280 h < sample times <706) in GSE77791 and GSE19743 were divided into two cohorts randomly to train and validate the regression model, the training cohort had 70% patients, and the validation cohort had 30%.

**FIGURE 8:**
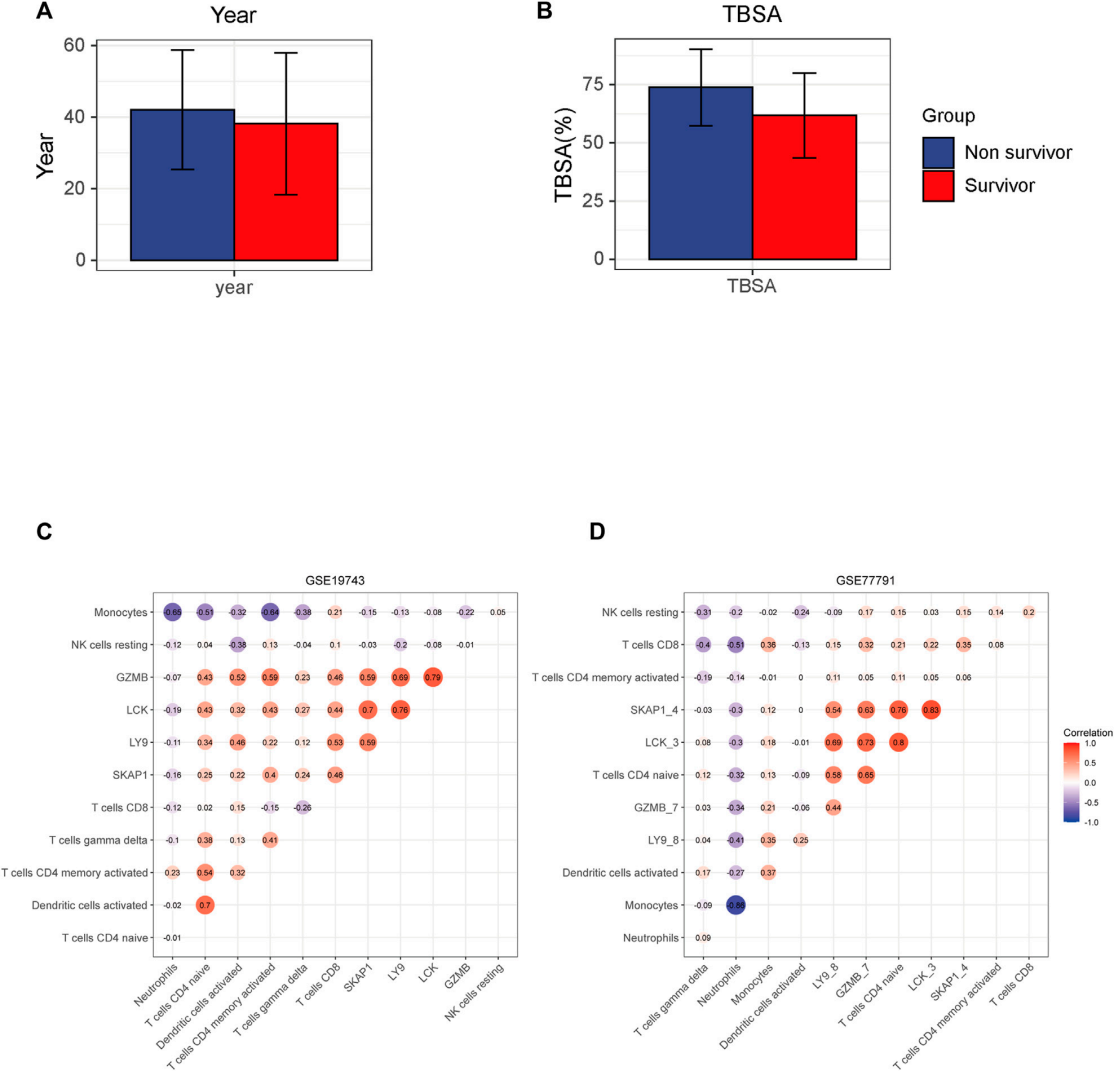
Results of the correlation between key genes and immune cells. **(A,B)** Different TBSA and years between survival and non-survival patients. Non-survival is older and has a larger TBSA. **(C,D)** Horizontal and ordinate are the names of the genes and cells. The figure in the circle means correlation and red means positive correlation and blue means negative.

26 variables were screened out in LASSO and logistic regression, and 7 genes that had AUC >0.6 were selected. Four immune-related genes LCK, SKAP1, GZMB, and LY9 were obtained by logistic regression modeling (Figures 7A–D). A nomogram plot was drawn to calculate the risk score of each patient, and the ROC curves were used to evaluate the prognostic ability of the risk score. AUC was 0.930 in the training cohort and AUC was 0.919 in the validation cohort (Figures 7E–G). The aclibration curve shows that the regression model has a good prediction ability (Figure 7H). 161patientswere randomly divided into two groups, training cohort (70%) and validation cohort (30%). Results showed that AUC^training^ = 0.946, AUC^validation^ = 0.902 (Figures 7I,J). The risk of non-survival was calculated by the nomogram containing risk score, age, and TBSA. AUC^risk score+TBSA+age^ = 0.945 > AUC^risk score^ = 0.933 (*p* < 0.05) (Figures 7K,L). Incorporating TBSA and age improves model predictive ability. Dead patients were older in age, had larger TBSA, and were not different in inhalation injury (Figures 8A,B) (Table 3). LCK, SKAP1, GZMB, and LY9 are associated with T cell CD4 naive, T cell CD4 memory activated, and T cell CD8 (Figures 8C,D).

**TABLE 3:** Differences between high- and low-risk burns that were divided by the regression model in inhalation injury.

Group	In inhalation injury		Value of chi-square	P
	Yes	No		
High risk	6	9	0.216	0.624
Low risk	14	28		

### Validation of Key Gene Expression

**FIGURE 9:**
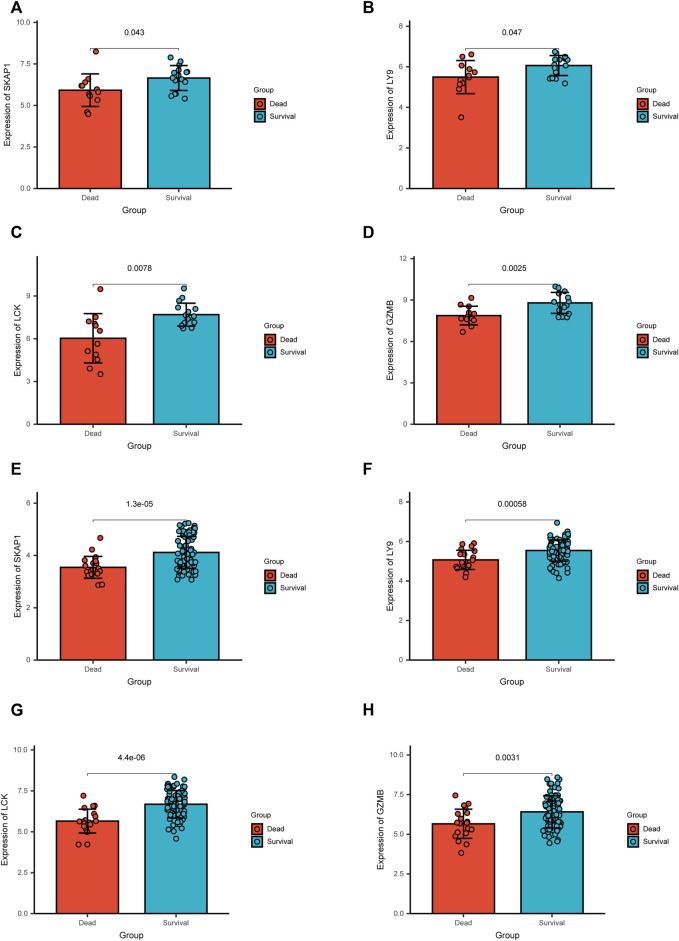
Results of the differential expression analysis of the microarray data in two independent cohorts.**(A–D)** In GSE19743, key genes, SKAP1, LY9, LCK, and GZMB, were up-regulated in survival patients. **(E–H)** In GSE77791, key genes, SKAP1, LY9, LCK, and GZMB, were up-regulated in survival patients.

In GSE19743 cohort, key genes, LY9, SKAP1, GZMB, and LCK, were highly expressed in survival patients. The same result was presented in GSE77791 (Figures 9A–H).

## Discussion

Physiological characteristics of the inflammatory response and immunosuppression in severe burns are the disorder of the number and proportion of immune cells. After severe burns, monocytes, macrophages, and neutrophils are activated. DAMP and PAMP recognize TLR to activate NF-κB. NF-κB is involved in the activation of inflammatory factors such as IL-1, IL-6, IL-8, IL-18, and TNF, resulting in a strong inflammatory response (D'Arpa and Leung 2017). Subsequently, immune function is inhibited. The antigen-presenting function of the macrophages and the killing function of the neutrophils are weakened, followed by the decreasing proliferation of T cells, particularly in the differentiation, proliferation, and function of Th cells (Miller et al., 2007). The main manifestation is the inhibition of Th1 cell differentiation, and a relative increase of Th2 cell differentiation leads to pro-inflammatory inhibition and anti-inflammatory activation. The changes in cytokines and cell proportion are not only the results of severe burns but also the important causes of immunosuppression and inflammatory response syndrome, which are related to the prognosis of patients and play an important role in the development of immunotherapy targets (Hur et al., 2015). In the GSVA results (Figure 2C), we found that the pathway of the T cell, and macrophage (negative regulated) were activated in the controls contrast to severe burns, and the pathways of the neutrophil and monocyte were inhibited, which may be an important reason for the activation of neutrophil, monocyte, and macrophages and the inhibition of T-cell function in severe burns. All the aforementioned data are consistent with the previous research. In addition, we also found that the pathways of B cells, NK cells, and T follicular-assistedpara-cellular were inhibited while dendritic cells were activated. These cells are the key to the immune response, but have not been revealed in the immunosuppression of severe burns.

The intersection of Cibersort and GSVA results showed that the disorder of immune cell subtypes in severe burns included T cell CD8, T cell CD4 naive, T cell CD4 memory resting, T cell CD4 memory activated, NK cell resting, monocytes, macrophages M0, dendritic cells resting, and neutrophils. Subtype disorders of immune cells are an important basis for immune dysfunction. Although the quantitative changes and pathway activation/inhibition of these subtypes have not been studied in the burn, they play an important role in the proliferation, differentiation, and function of T cells, NK cells, mononuclear macrophages, dendritic cells, and neutrophils. The decrease of CD4 naive T cells directly leads to the decrease of Th cells. In our study, we found that CD4 naive T cells decreased in severe burn patients, which could differentiate into Th1, Th2, Th17, and Treg cells (Zhou et al., 2009). In addition to the decrease in cell number, the proportion of different cell subtypes is also imbalanced. For example, Th1/Th2 decreased and Treg/Th17 increased in severe burns, which are important causes of adaptive immune dysfunction (O'Sullivan et al., 1995; MacConmara et al., 2011; Rendon and Choudhry 2012; Valvis et al., 2015). Our study found that CD8T cells and NK cells decreased in severe burns, which are principal cells against pathogens. In the early stages of burns, an inflammatory response will lead to an increase in the number of CD8 cells and NK cells, but a significant decrease will soon follow (Sherwood and Toliver-Kinsky 2004; Patil et al., 2016). Although the reason for the depletion of NK cells has not been found, excessive stress can lead to the depletion of CD8T cells, which may be the reason for the significant reduction of CD8T cells in the mid-burn stage (Sherwood and Toliver-Kinsky 2004; Kurachi 2019). Our study also found that monocytes, macrophages, dendritic cells, and neutrophils were significantly increased in patients with severe burns. The aforementioned cells were the key cells of the inflammatory response, connecting innate immunity, and adaptive immunity. Neutrophils, dendritic cells, and mononuclear macrophages are activated after a burn, causing a strong inflammatory response, which is an important cause of subsequent multiple organ failure and sepsis (Sherwood and Toliver-Kinsky 2004). In addition, over-activated neutrophils will also suppress the function of T cells and affect the adaptive immune response (Aarts et al., 2019). We believe that the changes of these cell subtypes play an important role in the immunosuppression of severe burns, and have a significant prognostic and therapeutic value. Therefore, we used WGCNA to analyze gene changes associated with these immune cells.

Considering that T cells are the main effector cells of adaptive immune response and play an important role in immunosuppression after burns, we selected modules correlated to T cells. Four modules containing differently expressed genes were found to be associated with cell subtypes in the WGCNA analysis (Figures 4A–D), with 748 genes related to the immune pathway (Figures 4E–H). The related pathways are mainly related to the immune-related gene functions and cell structures such as T cells, immune response, MHC II class protein complex, CD4 receptor, and Ca^2+^ signal pathway (Figures 5A–D). The main physiological manifestation of immunosuppression in patients with severe burns is a decrease in the adaptive immune response. T cells are key cells for adaptive immune response. Th1/Th2 ratio is an important factor in immune function. Th-1 produces IL-2 and IFN-γ and activates the immune response. Th-2 produces IL-4 and IL-10 and inhibits the immune response (Abbas et al., 1996). The Ca^2+^ signal pathway is associated with IL-2 production and plays an important role in immune function (Sayeed 1996). In addition, Th17 secretes IL-22 to active epithelial cells, participating in chemotaxis, tissue repair, and antimicrobial peptide expression to prevent bacterial invasion and epithelial cell proliferation and differentiation (Rendon and Choudhry 2012). This effect of Th-17 cells is important because severe burns can induce mucosal atrophy and apoptosis, as well as damage to the homeostasis of intestinal epithelial cells (Magnotti and Deitch 2005). Intestinal mucosal barrier is impaired as early as 5 minutes after severe burns, which increases the risk of bacterial translocation and sepsis. Th-17 cells have been proved to be able to prevent local and systemic proliferation of common infection sources after burning, such as *Bacteroides* fragileus, *Klebsiella pneumoniae*, and *Candida albicans* (Rendon and Choudhry 2012; Rani et al., 2014). T cells are not only an important manifestation of immunosuppression, but also an important therapeutic target for improving immune function. IL-15 treatment can improve the reduction of CD4 + T (Th) cells. Blocking CD47/CD172 signaling pathway can improve the reduction of CD4 + T cells and CD8 + T cells, thereby improving immune function (Patil et al., 2016; Beckmann et al., 2020). Flt3 ligand treatment can alleviate T-cell dysfunction and significantly improve the prognosis of septic mice. Therefore, we believe that T-cell-related genes play an important role in the development of T-cell function inhibition, have an important prognostic value, and are likely to be targeted for immunotherapy.

Among 210 immune-related genes in PPI network, 53 genes are hub genes (Figures 6A–D). Twenty-six genes were finally selected by LASSO regression, of which seven genes had significant indigenous prognosis (AUC > 0.7, *p* < 0.05), namely LCK, SKAP1, CX3CR1, FYN, GZMB, LY9, and FYN. The genes were incorporated into the logistic regression to construct the regression that included 4 key genes, LCK, SKAP1, GZMB, and LY9 (Figures 7A–D). In the GSE77791 (training cohort), AUC = 0.930 (Figure 7F), and calibration curve indicated that the model had an excellent prediction ability (Figure 7H). In the GSE19743 (validation cohort), AUC = 0.919 (Figure 7G). We included age and TBSA in the regression model for all patients (161), AUC^risk score+TBSA+age^ = 0.945 > AUC^risk score^ = 0.933 (Figure 7L). The prediction ability of the model was improved. In addition, there were significant differences in the burn area and age between survival and non-survival patients (Figures 8A,B). So, the prognostic function of our regression includes the effects of burn area and age.

LCK, SKAP1, GZMB, and LY9 are related to T cells, such as T cells CD4 naive, T cells CD4 memory activated, and T cells CD8. The correlation between gene expression and cell proportion indicates that genes may be potential biomarkers that characterize the number and function of cells.

The protein encoded by the LCK gene is a key molecule for differentiation and maturation of developing T cells. LCK exists in all normal T cells. In the cells, LCK is located in the plasma membrane and vesicles around the centrosome, which is related to the cytoplasmic tail of CD4 co-receptors on helper T cells and CD8 co-receptors on cytotoxic T cells, to help T-cell receptor (TCR) complexes signal and participate in the TCR-mediated T-cell activation (Shebzukhov et al., 2017). Human somatic cell experiments showed that the inhibition of LCK expression led to the inhibition of the TCR pathway, thereby hindering the differentiation and development of T cells. Targeted destruction of LCK can lead to T-cell development stagnation in the thymus (van Oers et al., 1996). Although there are few studies on the LCK gene in severe burns, the expression of the LCK gene is significantly related to T-cell subtypes, which is an important molecule to characterize the number and activity of T cells. In addition, therapies targeting LCK have been shown to promote/inhibit T-cell growth and development in a variety of diseases such as type 1 diabetes, colon cancer, asthma, and organ transplant rejection, thereby altering disease outcomes (Sabat et al., 2006; Gholamin et al., 2015). Therefore, LCK, depending on its correlation with T cells, is expected to provide a predictive value for T-cell function and become an important target for the treatment of T-cell dysfunction in severe burns.

SKAP1 gene encodes T-cell adaptor protein which is a key regulator of TCR-mediated LFA-1 signaling (inside-out/outside-in signaling), T-cell receptor-induced activation of LFA-1 to promote T-cell adhesion and interaction with antigen-presenting cells (APCs) (Witte et al., 2017). SKAP1 deficiency affects TCR activation (Lim et al., 2016). The expression of SKAP1 was correlated with T-cell function and disease development. In SKAP1 deficient mice, it was found that IL17 cytokines decreased and T-cell infiltration decreased, which alleviated collagen-induced osteoarthritis (Smith et al., 2016). Th17 is an important mucosal immune cell, which has an important relationship with intestinal flora translocation after burns. SKAP1 deficiency may lead to Th17 deficiency and promote the development of the disease. Although SKAP1 has not been studied in immunosuppression after burns, we believe that SKAP1 can characterize T-cell function and is a promising immunotherapy target for improving T-cell function.

GZMB encodes Granzyme B which is mainly secreted by natural killer (NK) cells and cytotoxic T lymphocytes (CTLs). Granzyme B induces target cell apoptosis and can impact processes such as tissue remodeling, barrier function, autoantigen generation, and angiogenesis. It plays an important role in wound healing, chronic inflammation, and scar formation (Śledzińska et al., 2020). Therefore, the expression of GZMB reflects the differentiation of T-cell subtypes to some extent. LY9 encodes a homocellular surface receptor that exists on all thymocytes and is highly expressed on innate lymphocytes such as iNKT cells. LY9 plays an important role in maintaining T-cell subtype differentiation. The level of IL-4 in LY9-deficient mice was significantly increased, and IL-4 was mainly secreted by Th2 cells, which inhibited the inflammation and immune responses (Cuenca et al., 2018). In our experiment, we found that LY9 was significantly down-regulated, which may be one of the molecular mechanisms of the Th cell subtype disorder. Although GZMB and LY9 have not been further studied in the immunosuppression of severe burns, the proteins encoded by GZMB and LY9 play an important role in T-cell immune function, T-cell subtype differentiation, and wound healing. Obviously, GZMB and LY9 can be used as prognostic factors which can characterize physiological changes after severe burns.

Key genes have great potential in post-burn immunosuppression, which will be a meaningful research direction. In addition, in the differential expression analysis, it was found that in the two independent cohorts, the expression of key genes in survival patients was significantly increased (Figures 9A–H), which may indicate that the down-regulation of key genes is an important factor leading to immunosuppression and death, which needs further research.

Our experiment is the first to use WGCNA, GSVA, and LASSO regression to construct a gene prognosis model with genes in three severe burns cohorts (185 patients). In contrast to prognostic models for platelets, coagulation disorders, IFN-γ, IL-2, IL-4, Burn Severity Index (ABSI) score, Ryan score, Belgium Outcome Burn Injury (BOBI) score, and modified Baux score, our prognostic model was based on gene expression profile, which had a higher accuracy and was more convenient for clinical operation (Hur et al., 2015; Lip et al., 2019; Geng et al., 2020). Others use bioinformatics methods to study the pathophysiology of severe burns, but most are limited to animal models or have unstable and inaccurate prognostic indicators (Li et al., 2016; Fang et al., 2020; Liu et al., 2021). We first introduced WGCNA, CIBERSORT, GSVA, and LASSO into the analysis to find prognostic factors from the pathophysiological mechanism of immunosuppression after severe burns, so our prognostic model is more stable and reliable. In addition, we found that key genes were associated with immunosuppression after severe burns and were related to the ratio of specific immune cell types, which provided an important direction for the future development of immunotherapy targets. Of course, our experiment is also insufficient. We need to collect more information about patients, such as whether shock resuscitation or sepsis occurs, to further stabilize our model. Nevertheless, compared with other clinical prognosis models, our model showed a good prognosis ability in collaboration with age and burn area, and the gene expression and prognosis model were verified multi-dimensionally (three large cohorts, sequencing datasets, and multiple groupings).

## Summary

Our study found that immunosuppressive-related genes after severe burns had important prognostic value. The prognostic ability of LCK, SKAP1, GZMB, and LY9 in the gene expression profiles of 185 severe burns was superior to the current prognostic models and scale score.

## Data Availability

The original contributions presented in the study are included in the article/Supplementary Material, further inquiries can be directed to the corresponding author.

## Abbreviations

DAMP, damage-associated molecular patterns; GSVA, gene set variation analysis; GO, Gene Ontology; KEGG, Kyoto Encyclopedia of Genes and Genomes; KICs, key immune cells; LASSO, Least Absolute Shrinkage and Selectionator Operator; PPI, protein–protein interaction; PAMP, pathogen-associated molecular patterns; TBSA, total burn surface area; and WGCNA, weighted gene co-expression network analysis.
